# Supplementary material for: Resonant catalysis of thermally activated chemical reactions with vibrational polaritons
Source: Nat Commun. 2019 Oct 15;10:4685. doi: 10.1038/s41467-019-12636-1 (PMC6794294; doi:10.1038/s41467-019-12636-1)
Supplement: Supplementary file 1 — Supplementary Information [file 41467_2019_12636_MOESM1_ESM.pdf]

Supplementary Information for
Resonant catalysis of chemical reactions with vibrational polaritons

Campos-Gonzalez-Angulo et al.

SUPPLEMENTARY NOTES

Supplementary Note 1.

Here, we show the relation between reactant and product harmonic oscillator operators. Let us consider the vibrational Hamiltonians for the single-molecule reactant and product electronic states (we omit label (i) for simplicity hereafter),

$$\hat{H}_{\mathrm{R}} = \frac{\hat{p}^2}{2m} + \frac{m\omega_{\mathrm{R}}^2\hat{x}^2}{2} = \hbar\omega_{\mathrm{R}}\left(\hat{a}_{\mathrm{R}}^\dagger\hat{a}_{\mathrm{R}} + \frac{1}{2}\right), \tag{1}$$

$$\hat{H}_{\mathrm{P}} = \frac{\hat{p}^2}{2m} + \frac{m\omega_{\mathrm{P}}^2(\hat{x}-d_{\mathrm{P}})^2}{2} + \Delta E = \hbar\omega_{\mathrm{P}}\left(\hat{a}_{\mathrm{P}}^\dagger\hat{a}_{\mathrm{P}} + \frac{1}{2}\right) + \Delta E, \tag{2}$$

where m is the reduced mass of the mode, ω_A is the frequency of the mode in each electronic state ($A = \mathrm{R}, \mathrm{P}$), d_{P} is the difference between nuclear equilibrium configurations, ΔE is the energy difference between the electronic states, and $\hat{p}$ and $\hat{x}$ are the momentum and position operators for the described mode; therefore, the harmonic oscillator potential energy surface for P is a displaced-distorted version of that for R. The creation and annihilation operators are defined in terms of position and momentum ($d_{\mathrm{R}} = 0$),

$$\begin{aligned}\hat{a}_A^\dagger &= \sqrt{\frac{\omega_A m}{2\hbar}}\,(\hat{x}-d_A) - \frac{i\hat{p}}{\sqrt{2\hbar\omega_A m}},\\ \hat{a}_A &= \sqrt{\frac{\omega_A m}{2\hbar}}\,(\hat{x}-d_A) + \frac{i\hat{p}}{\sqrt{2\hbar\omega_A m}};\end{aligned} \tag{3}$$

conversely, the position-momentum representation is written in terms of the creation and annihilation operators as

$$\begin{aligned}\hat{x} - d_A &= \sqrt{\frac{\hbar}{2\omega_A m}}\left(\hat{a}_A^\dagger + \hat{a}_A\right),\\ \hat{p} &= \sqrt{\frac{\hbar\omega_A m}{2}}\,i\left(\hat{a}_A^\dagger - \hat{a}_A\right).\end{aligned} \tag{4}$$

Equation (4) implies

$$\begin{aligned}\frac{\hat{a}_{\mathrm{R}}^\dagger + \hat{a}_{\mathrm{R}}}{\sqrt{\omega_{\mathrm{R}}}} &= \frac{\hat{a}_{\mathrm{P}}^\dagger + \hat{a}_{\mathrm{P}} + \tilde{d}_{\mathrm{P}}}{\sqrt{\omega_{\mathrm{P}}}},\\ \sqrt{\omega_{\mathrm{R}}}\left(\hat{a}_{\mathrm{R}}^\dagger - \hat{a}_{\mathrm{R}}\right) &= \sqrt{\omega_{\mathrm{P}}}\left(\hat{a}_{\mathrm{P}}^\dagger - \hat{a}_{\mathrm{P}}\right),\end{aligned} \tag{5}$$

where $\tilde{d}_{\mathrm{P}} = \sqrt{2m/\hbar}\; d_{\mathrm{P}}$; therefore, the reactant operators are written in terms of product ones as

$$\begin{aligned}\hat{a}_{\mathrm{R}}^\dagger &= \frac{1}{2}\left(\sqrt{\frac{\omega_{\mathrm{R}}}{\omega_{\mathrm{P}}}} + \sqrt{\frac{\omega_{\mathrm{P}}}{\omega_{\mathrm{R}}}}\right)\hat{a}_{\mathrm{P}}^\dagger + \frac{1}{2}\left(\sqrt{\frac{\omega_{\mathrm{R}}}{\omega_{\mathrm{P}}}} - \sqrt{\frac{\omega_{\mathrm{P}}}{\omega_{\mathrm{R}}}}\right)\hat{a}_{\mathrm{P}} + \sqrt{\frac{\omega_{\mathrm{R}}}{\omega_{\mathrm{P}}}}\frac{\tilde{d}_{\mathrm{P}}}{2},\\ \hat{a}_{\mathrm{R}} &= \frac{1}{2}\left(\sqrt{\frac{\omega_{\mathrm{R}}}{\omega_{\mathrm{P}}}} - \sqrt{\frac{\omega_{\mathrm{P}}}{\omega_{\mathrm{R}}}}\right)\hat{a}_{\mathrm{P}}^\dagger + \frac{1}{2}\left(\sqrt{\frac{\omega_{\mathrm{R}}}{\omega_{\mathrm{P}}}} + \sqrt{\frac{\omega_{\mathrm{P}}}{\omega_{\mathrm{R}}}}\right)\hat{a}_{\mathrm{P}} + \sqrt{\frac{\omega_{\mathrm{R}}}{\omega_{\mathrm{P}}}}\frac{\tilde{d}_{\mathrm{P}}}{2}.\end{aligned} \tag{6}$$

These transformations can be written in terms of a squeezing and a displacement operator [1]:

$$\hat{\mathcal{S}}_{\mathrm{P}}(r) = \exp\left[\frac{r}{2}(\hat{a}_{\mathrm{P}}^2 - \hat{a}_{\mathrm{P}}^{\dagger 2})\right], \tag{7}$$

$$\hat{\mathcal{D}}_{\mathrm{P}}(\alpha) = \exp\left[\alpha(\hat{a}_{\mathrm{P}}^\dagger - \hat{a}_{\mathrm{P}})\right], \tag{8}$$

with actions given by

$$\hat{\mathcal{S}}_{\mathrm{P}}^\dagger(r)\hat{a}_{\mathrm{P}}^\dagger\hat{\mathcal{S}}_{\mathrm{P}}(r) = \hat{a}_{\mathrm{P}}^\dagger\cosh r - \hat{a}_{\mathrm{P}}\sinh r, \tag{9}$$

$$\hat{\mathcal{D}}_{\mathrm{P}}^\dagger(\alpha)\hat{a}_{\mathrm{P}}\hat{\mathcal{D}}_{\mathrm{P}}(\alpha) = \hat{a}_{\mathrm{P}} + \alpha. \tag{10}$$

Therefore,

$$\hat{a}_{\mathrm{R}}^{\dagger} = \hat{\mathcal{D}}_{\mathrm{P}}^{\dagger}(\alpha)\hat{\mathcal{S}}_{\mathrm{P}}^{\dagger}(r)\hat{a}_{\mathrm{P}}^{\dagger}\hat{\mathcal{S}}_{\mathrm{P}}(r)\hat{\mathcal{D}}_{\mathrm{P}}(\alpha)$$
$$\hat{a}_{\mathrm{R}} = \hat{\mathcal{D}}_{\mathrm{P}}^{\dagger}(\alpha)\hat{\mathcal{S}}_{\mathrm{P}}^{\dagger}(r)\hat{a}_{\mathrm{P}}\hat{\mathcal{S}}_{\mathrm{P}}(r)\hat{\mathcal{D}}_{\mathrm{P}}(\alpha), \tag{11}$$

for

$$r = \ln\sqrt{\frac{\omega_{\mathrm{R}}}{\omega_{\mathrm{P}}}}, \tag{12}$$

$$\alpha = \tilde{d}_{\mathrm{P}}. \tag{13}$$

Supplementary Note 2.

Here, we introduce the initial and final many-body vibronic states. The rate to calculate corresponds to the stoichiometric process

$$(M-N)\mathrm{R} + N\mathrm{P} \longrightarrow (M-N-1)\mathrm{R} + (N+1)\mathrm{P}, \tag{14}$$

where N is the number of molecules in the product electronic state P, and $M-N$ is the number of molecules in the reactant electronic state R, such that M is the total number of molecules in the reaction vessel. Assigning labels to each molecule, without loss of generality, the transformation of the $N+1$-th molecule can be written in the form

$$\sum_{i=N+1}^{M}\mathrm{R}_i + \sum_{j=1}^{N}\mathrm{P}_j \longrightarrow \sum_{i=N}^{M}\mathrm{R}_i + \sum_{j=1}^{N+1}\mathrm{P}_j, \tag{15}$$

which reduces to

$$\mathrm{R}_{N+1} \longrightarrow \mathrm{P}_{N+1}. \tag{16}$$

The charge transfer is ruled by the adiabatic coupling $\hat{J} = J_{\mathrm{RP}}\sum_{i=1}^{M}\left(|\mathrm{R}_i\rangle\langle\mathrm{P}_i| + |\mathrm{P}_i\rangle\langle\mathrm{R}_i|\right)$; then, the matrix element that describes the process of our focus is

$$\langle M-N, N|\hat{J}|M-N-1, N+1\rangle = J_{\mathrm{RP}}\langle M-N, N|\mathrm{R}_{N+1}\rangle\langle\mathrm{P}_{N+1}|M-N-1, N+1\rangle, \tag{17}$$

with many-body vibronic states given by

$$|X, Y\rangle = |\mathrm{P}_1\mathrm{P}_2\ldots\mathrm{P}_{Y-1}\mathrm{P}_Y\mathrm{R}_{Y+1}\mathrm{R}_{Y+2}\ldots\mathrm{R}_{X+Y-1}\mathrm{R}_{X+Y}\rangle \otimes |\Phi_X^Y\rangle, \tag{18}$$

where $|\Phi_X^Y\rangle$ is an eigenfunction of a vibrational Hamiltonian of the form

$$\begin{aligned}\hat{H}_{X,Y} &= \hat{H}_{\mathrm{ph}} + \sum_{i=1}^{Y}\left(\hat{H}_{\mathrm{P}}^{(i)} + \hat{\mathcal{V}}_{\mathrm{int}}^{(i)}\right) + \sum_{j=Y+1}^{X+Y}\hat{H}_{\mathrm{R}}^{(j)} \\ &= \hat{H}_{+(Y)} + \hat{H}_{-(Y)} + \sum_{k=1}^{Y-1}\hat{H}_{\mathrm{D}(Y)}^{(k)} + \sum_{i=1}^{Y}\hat{H}_{\mathrm{S}}(\hat{\mathbf{q}}_{\mathrm{S}}^{(i)}) + Y\Delta E + \sum_{j=Y+1}^{X+Y}\hat{H}_{\mathrm{R}}^{(j)}.\end{aligned} \tag{19}$$

In Supplementary equation (19), we have used the notation introduced in equation (2), and $\hat{H}_{\pm(Y)} = \hbar\omega_{\pm}\left(\hat{a}_{\pm(Y)}^{\dagger}\hat{a}_{\pm(Y)} + \frac{1}{2}\right)$ and $\hat{H}_{\mathrm{D}(Y)}^{(k)} = \hbar\omega_{\mathrm{P}}\left(\hat{a}_{\mathrm{D}(Y)}^{\dagger(k)}\hat{a}_{\mathrm{D}(Y)}^{(k)} + \frac{1}{2}\right)$ are the Hamiltonians of the upper/lower and k-th dark modes, respectively, all with creation and annihilation operators as defined in equation (3). Therefore, the matrix element corresponding to the transition becomes

$$\langle M-N, N|\hat{J}|M-N-1, N+1\rangle = J_{\mathrm{RP}}\langle\Phi_{M-N}^{N}|\Phi_{M-N-1}^{N+1}\rangle. \tag{20}$$

Supplementary Note 3.

Here, we derive the tridimensional Franck-Condon factor in equation (12) of the main text. The non-vanishing overlaps between the vibrational ground state of the reactants and an arbitrary vibrational excitation with quantum numbers $\{v_+, v_-, v_\mathrm{D}\}$ on the products can be written in terms of creation operators as

$$\left\langle 0_0 0_{\mathrm{B}(N-1)} 0'_N \mid v_+ v_- v_\mathrm{D} \right\rangle = \left\langle 0_0 0_{\mathrm{B}(N-1)} 0'_N \right| \frac{\left(\hat{a}^\dagger_{+(N)}\right)^{v_+}}{\sqrt{v_+!}} \frac{\left(\hat{a}^\dagger_{-(N)}\right)^{v_-}}{\sqrt{v_-!}} \frac{\left(\hat{a}^{(N-1)\dagger}_\mathrm{D}\right)^{v_\mathrm{D}}}{\sqrt{v_\mathrm{D}!}} \left| 0_{+(N)} 0_{-(N)} 0^{(N-1)}_\mathrm{D} \right\rangle . \tag{21}$$

These operators acting in the UP and LP can be written as linear combinations of the operators acting on the electromagnetic mode and the bright mode [equation (3)], i.e.,

$$\begin{aligned}\left\langle 0_0 0_{\mathrm{B}(N-1)} 0'_N \mid v_+ v_- v_\mathrm{D} \right\rangle = \left\langle 0_0 0_{\mathrm{B}(N-1)} 0'_N \right| &\frac{\left(\hat{a}^\dagger_0 \cos\theta - \hat{a}^\dagger_{\mathrm{B}(N)} \sin\theta\right)^{v_+}}{\sqrt{v_+!}} \frac{\left(\hat{a}^\dagger_0 \sin\theta + \hat{a}^\dagger_{\mathrm{B}(N)} \cos\theta\right)^{v_-}}{\sqrt{v_-!}} \\ &\times \frac{\left(\hat{a}^{(N-1)\dagger}_\mathrm{D}\right)^{v_\mathrm{D}}}{\sqrt{v_\mathrm{D}!}} \left| 0_0 0_{\mathrm{B}(N)} 0^{(N-1)}_\mathrm{D} \right\rangle\end{aligned} \tag{22}$$

The binomial theorem yields

$$\begin{aligned}\left\langle 0_0 0_{\mathrm{B}(N-1)} 0'_N \mid v_+ v_- v_\mathrm{D} \right\rangle = \sum_{m=0}^{v_+} \sum_{n=0}^{v_-} \binom{v_+}{m} \binom{v_-}{n} \left\langle 0_0 0_{\mathrm{B}(N-1)} 0'_N \right| &\frac{\left(\hat{a}^\dagger_0 \cos\theta\right)^m \left(-\hat{a}^\dagger_{\mathrm{B}(N)} \sin\theta\right)^{v_+ - m}}{\sqrt{v_+!}} \\ \times \frac{\left(\hat{a}^\dagger_0 \sin\theta\right)^n \left(\hat{a}^\dagger_{\mathrm{B}(N)} \cos\theta\right)^{v_- - n}}{\sqrt{v_-!}} &\frac{\left(\hat{a}^{(N-1)\dagger}_\mathrm{D}\right)^{v_\mathrm{D}}}{\sqrt{v_\mathrm{D}!}} \left| 0_0 0_{\mathrm{B}(N)} 0^{(N-1)}_\mathrm{D} \right\rangle .\end{aligned} \tag{23}$$

Since $[\hat{a}_0, \hat{a}_{\mathrm{B}(N)}] = 0$, the only non-vanishing terms are those with $m = n = 0$, otherwise the overlap in the photonic mode would be between non-displaced states with different excitations; therefore,

$$\left\langle 0_0 0_{\mathrm{B}(N-1)} 0'_N \mid v_+ v_- v_\mathrm{D} \right\rangle = \left\langle 0_{\mathrm{B}(N-1)} 0'_N \right| \frac{\left(-\hat{a}^\dagger_{\mathrm{B}(N)} \cos\theta\right)^{v_+}}{\sqrt{v_+!}} \frac{\left(\hat{a}^\dagger_{\mathrm{B}(N)} \sin\theta\right)^{v_-}}{\sqrt{v_-!}} \frac{\left(\hat{a}^{(N-1)\dagger}_\mathrm{D}\right)^{v_\mathrm{D}}}{\sqrt{v_\mathrm{D}!}} \left| 0_{\mathrm{B}(N)} 0^{(N-1)}_\mathrm{D} \right\rangle . \tag{24}$$

Moreover, the creation operators acting on the bright and dark modes can be expressed as linear combinations of operators acting on the N-th molecule and the bright mode that excludes it [equation (6)], i.e.,

$$\begin{aligned}\left\langle 0_0 0_{\mathrm{B}(N-1)} 0'_N \mid v_+ v_- v_\mathrm{D} \right\rangle = \frac{(-\cos\theta)^{v_+} (\sin\theta)^{v_-}}{\sqrt{v_+! v_-! v_\mathrm{D}!}} &\left\langle 0_{\mathrm{B}(N-1)} 0'_N \right| \left(\hat{a}^\dagger_{\mathrm{B}(N-1)} \sqrt{\tfrac{N-1}{N}} + \hat{a}^\dagger_N \sqrt{\tfrac{1}{N}}\right)^{v_+} \\ \times \left(\hat{a}^\dagger_{\mathrm{B}(N-1)} \sqrt{\tfrac{N-1}{N}} + \hat{a}^\dagger_N \sqrt{\tfrac{1}{N}}\right)^{v_-} &\left(\hat{a}^\dagger_{\mathrm{B}(N-1)} \sqrt{\tfrac{1}{N}} - \hat{a}^\dagger_N \sqrt{\tfrac{N-1}{N}}\right)^{v_\mathrm{D}} \left| 0_{\mathrm{B}(N-1)} 0_N \right\rangle .\end{aligned} \tag{25}$$

By expanding the binomials as before, and discarding the terms that excite the $\mathrm{B}(N-1)$ mode, we arrive at

$$\begin{aligned}\left\langle 0_0 0_{\mathrm{B}(N-1)} 0'_N \mid v_+ v_- v_\mathrm{D} \right\rangle &= \frac{(-\cos\theta)^{v_+} (\sin\theta)^{v_-}}{\sqrt{v_+! v_-! v_\mathrm{D}!}} \left\langle 0'_N \right| \left(-\hat{a}^\dagger_N \sqrt{\tfrac{1}{N}}\right)^{v_+} \left(\hat{a}^\dagger_N \sqrt{\tfrac{1}{N}}\right)^{v_-} \left(-\hat{a}^\dagger_N \sqrt{\tfrac{N-1}{N}}\right)^{v_\mathrm{D}} \left| 0_N \right\rangle \\ &= \frac{1}{\sqrt{v_+! v_-! v_\mathrm{D}!}} \left(-\frac{\cos\theta}{\sqrt{N}}\right)^{v_+} \left(\frac{\sin\theta}{\sqrt{N}}\right)^{v_-} \left(-\sqrt{\frac{N-1}{N}}\right)^{v_\mathrm{D}} \left\langle 0'_N \right| \left(\hat{a}^\dagger_N\right)^{v_+ + v_- + v_\mathrm{D}} \left| 0_N \right\rangle .\end{aligned} \tag{26}$$

Acting the creation operator on the N-th mode allows us to write

$$\left\langle 0_0 0_{\mathrm{B}(N-1)} 0'_N \mid v_+ v_- v_\mathrm{D} \right\rangle = \sqrt{\frac{(v_+ + v_- + v_\mathrm{D})!}{v_+! v_-! v_\mathrm{D}!}} \left(-\frac{\cos\theta}{\sqrt{N}}\right)^{v_+} \left(\frac{\sin\theta}{\sqrt{N}}\right)^{v_-} \left(-\sqrt{\frac{N-1}{N}}\right)^{v_\mathrm{D}} \left\langle 0'_N \mid (v_+ + v_- + v_\mathrm{D})_N \right\rangle . \tag{27}$$

Therefore, the square of the Franck-Condon factor in equation (21) is

$$\left| \left\langle 0_0 0_{\mathrm{B}(N-1)} 0'_N \mid v_+ v_- v_\mathrm{D} \right\rangle \right|^2 = \binom{v_+ + v_- + v_\mathrm{D}}{v_+, v_-, v_\mathrm{D}} \left(\frac{\cos^2\theta}{N}\right)^{v_+} \left(\frac{\sin^2\theta}{N}\right)^{v_-} \left(\frac{N-1}{N}\right)^{v_\mathrm{D}} \left| \left\langle 0'_N \mid (v_+ + v_- + v_\mathrm{D})_N \right\rangle \right|^2 . \tag{28}$$

Supplementary Note 4.

Here, we discuss the integration of the rate law.

Chemical Master Equation. The chemical master equation for the reaction in equation (7) is given by

$$\frac{\partial}{\partial t}\Pr(N_{\mathrm{R}}, t \mid M, 0) = a(N_{\mathrm{R}}+1)\Pr(N_{\mathrm{R}}+1, t \mid M, 0) - a(N_{\mathrm{R}})\Pr(N_{\mathrm{R}}, t \mid M, 0), \tag{29}$$

where $\Pr(n, t \mid m, 0)$ is the conditional probability to observe n molecules of the donor at time t given that there were m at $t = 0$, and $a(n) = nk^{VSC}_{R\to P}(n)$ is the propensity function [2]. Since $\Pr(M+1, t \mid M, 0) \equiv 0$, this equation can be solved exactly by successively plugging $N_{\mathrm{R}} = M, M-1, \ldots, 0$, yielding

$$\Pr(M-n, t \mid M, 0) = (-1)^n \prod_{i=0}^{n-1} a(M-i) \sum_{j=0}^{n} \frac{\mathrm{e}^{-a(M-j)t}}{\prod_{\ell=0}^{n}\left[a(M-j) - a(M-\ell) + \delta_{j\ell}\right]}. \tag{30}$$

This probability density function can be used to determine the average number of donor molecules at a given time:

$$\langle N_{\mathrm{R}}(t)\rangle \doteq \sum_{n=0}^{M}(M-n)\Pr(M-n, t \mid M, 0). \tag{31}$$

Taking the time derivative of this average yields equation (18).

However, for the number of molecules considered, $M = 10^7$, this calculation becomes intractable; therefore, we resort to the strategy described in the Materials and Methods section of the main manuscript and corroborate its validity with the stochastic simulation algorithm [2].

Stochastic Simulation Algorithm (SSA). For the decomposition reaction in equation (18), we can define

$$p(\tau \mid M-n, t) = a(M-n)\exp\left[-a(M-n)\tau\right], \tag{32}$$

as the conditioned probability density function for the time of the next reaction (τ) given that the number of donor molecules left is $M-n$ at t. This function enables the construction of an exact numerical realization of the reaction with the following algorithm:

1. Initialize the system at $N_{\mathrm{R}}(0) = M$.
2. With the system in state $N_{\mathrm{R}}(t) = M - n(t)$, evaluate $a(N_{\mathrm{R}})$.
3. Generate a value for $\tau = -\ln(r)/a(N_{\mathrm{R}})$, where R is a uniformly distributed random number.
4. Perform the next reaction by making $N_{\mathrm{R}}(t+\tau) = N_{\mathrm{R}}(t) - 1$.
5. Register $N_{\mathrm{R}}(t)$ as needed. Return to 2 or else end the simulation.

In Supplementary Table 1, we show the correlation (r^2) between the reaction times calculated according to the mean-field finite difference approach described in the manuscript and the reaction times corresponding to the same step in the reaction with populations obtained from the mean of 100 trajectories computed with the SSA algorithm. Since these correlations are very close to the unity, we conclude that the compared methods are numerically equivalent [3]. These observations are consistent with a recent study that shows that mean-field theories provide good descriptions for polaritonic systems involving a large number of molecules [4].

Supplementary Table 1.

Ω	$\Delta/\omega_{\mathrm{P}}$	r^2
0	-	0.9970
≥ 0	–0.02	0.9965
	0	0.9982
	0.02	0.9973
	0.04	0.9970
	0.06	0.9969

[1] D. F. Walls and G. J. Milburn, *Quantum Optics* (Springer Berlin Heidelberg, 2008).
[2] D. T. Gillespie, Annu Rev Phys Chem **58**, 35 (2007).
[3] D. T. Gillespie, J Phys Chem B **113**, 1640 (2009).
[4] J. Keeling and P. G. Kirton, Phys. Rev. A **97**, 053836 (2018).
[5] A. Strashko and J. Keeling, Phys Rev A **94**, 023843 (2016).

SUPPLEMENTARY FIGURES

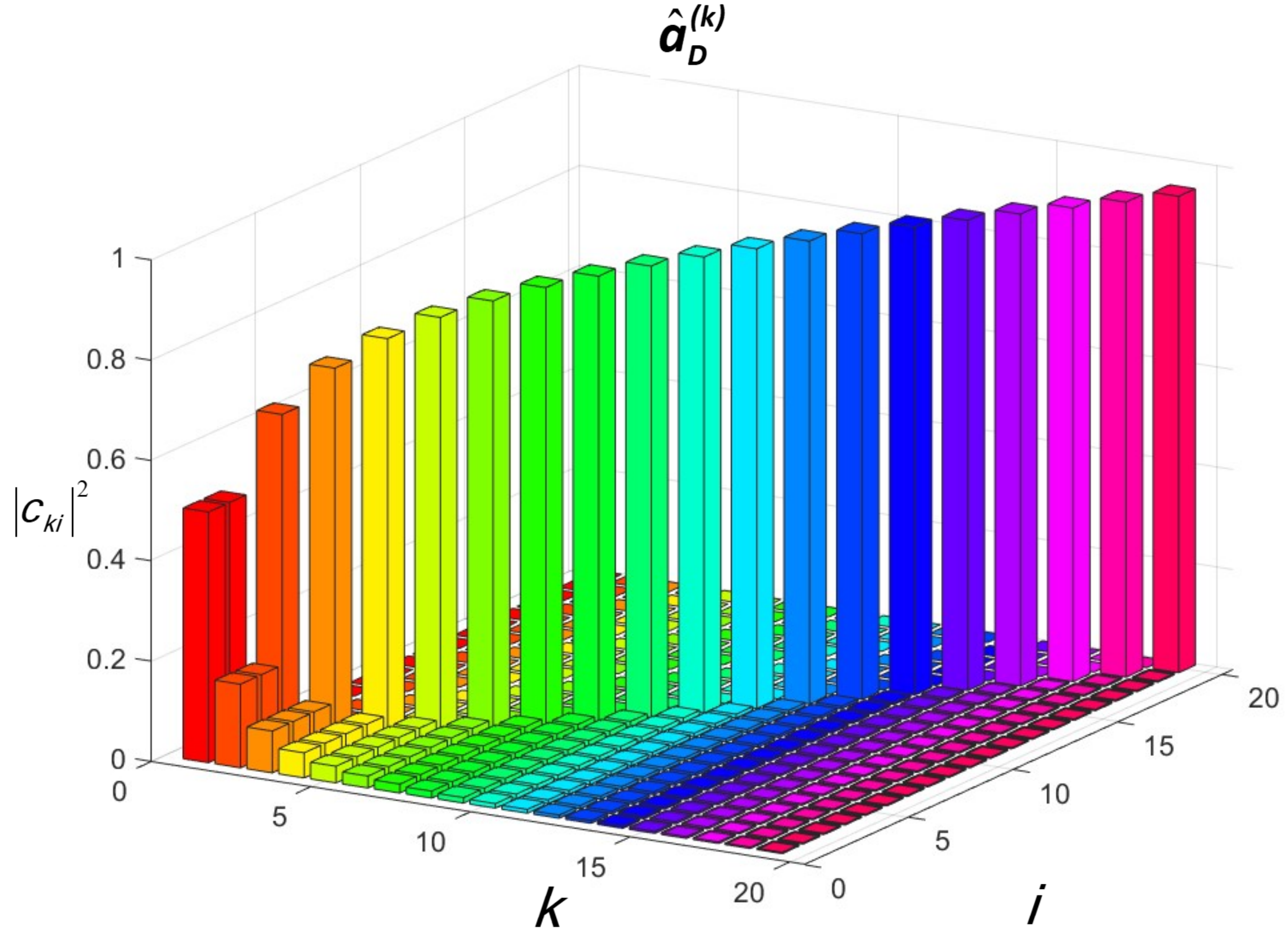

Supplementary Fig. 1. Probability coefficients for each molecular mode in the quasi-localized basis of dark modes defined in equation (6). As the dark mode index, k, increases, it becomes more localized in the k-th molecule, leaving a long tail behind it [5].

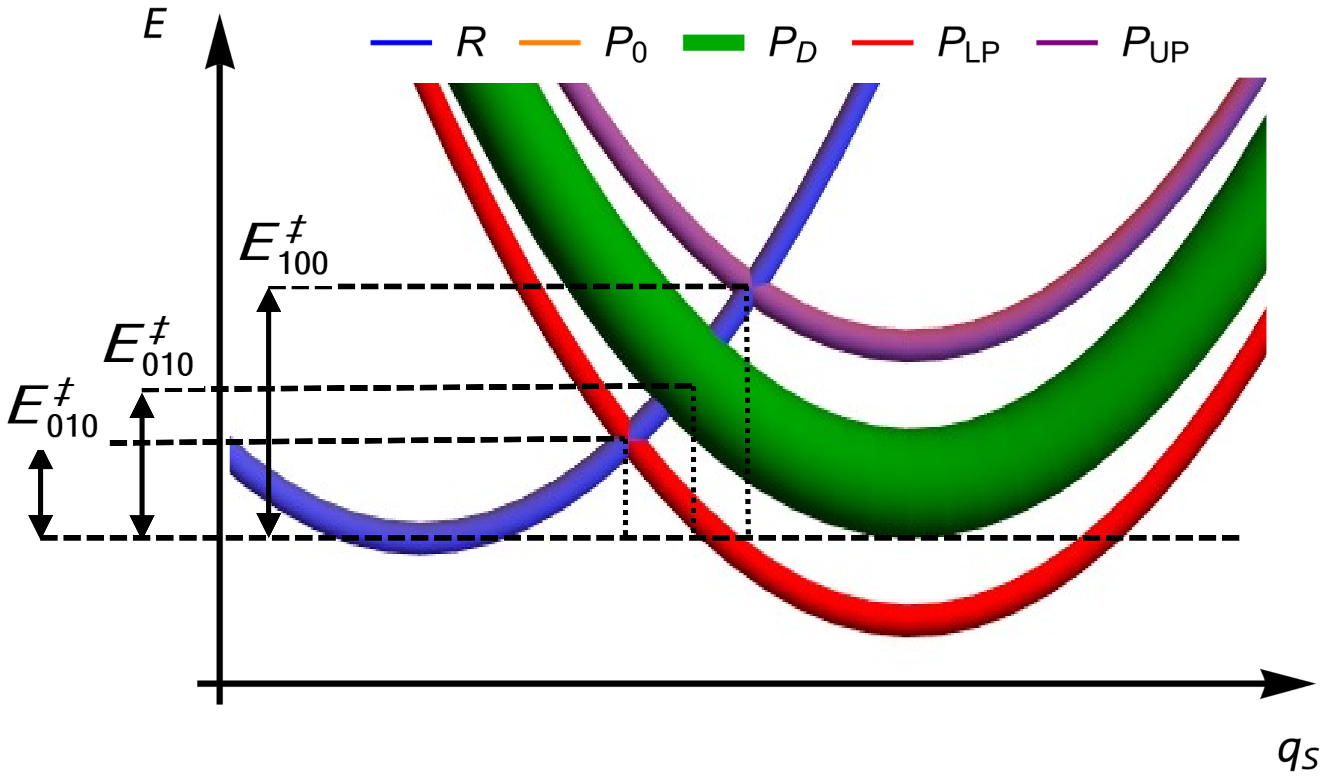

Supplementary Fig. 2. Amplification of Fig. 3, showing a situation where a polariton channel dominates the kinetics of a reaction starting at reactant R. The channel involving a vibrational excitation in the lower polariton of the product (P_{LP}) features a small enough activation barrier $E^{\ddagger}_{001}$ that can effectively compete against the many channels ending with a vibrational excitation in any of the dark modes, P_D, which feature corresponding activation energies $E^{\ddagger}_{001}$. These two activation energies are much smaller than $E^{\ddagger}_{000}$, the one associated with the channel leading to the global ground state of the products, P_0 (not shown in this amplified figure).
